# *Andrographis paniculata* and Its Bioactive Diterpenoids Protect Dermal Fibroblasts against Inflammation and Oxidative Stress

**DOI:** 10.3390/antiox9050432

**Published:** 2020-05-15

**Authors:** Eugenie Mussard, Sundy Jousselin, Annabelle Cesaro, Brigitte Legrain, Eric Lespessailles, Eric Esteve, Sabine Berteina-Raboin, Hechmi Toumi

**Affiliations:** 1 Laboratory I3MTO, EA 4708, Université d’Orléans, CEDEX 2, 45067 Orléans, France; eugenie.mussard@univ-orleans.fr (E.M.); sundy.jousselin@univ-orleans.fr (S.J.); annabelle.cesaro@univ-orleans.fr (A.C.); eric.lespessailles@chr-orleans.fr (E.L.); 2 NOVAXIA-6 Rue des Champs Godin, 41220 St Laurent Nouan, France; b.legrain@labo-novaxia.com; 3 Service de Rhumatologie, Centre Hospitalier Régional d’Orléans CHRO, 14 Avenue de l’Hôpital, 45100 Orléans, France; 4 Plateforme Recherche Innovation Médicale Mutualisée d’Orléans, Centre Hospitalier Régional d’Orléans 14 Avenue de l’Hôpital, 45100 Orléans, France; 5 Service de Dermatologie, Centre Hospitalier Régional d′Orléans 14 Avenue de l’Hôpital, 45100 Orléans, France; eric.esteve@chr-orleans.fr; 6 Institut de Chimie Organique et Analytique ICOA, Université d’Orléans-Pôle de Chimie, UMR CNRS 7311, Rue de Chartres-BP 6759, CEDEX 2, 45067 Orléans, France; sabine.berteina-raboin@univ-orleans.fr

**Keywords:** *Andrographis paniculata*, dermal fibroblast, inflammation, oxidative stress, skin aging, biomolecules, natural products

## Abstract

*Andrographis paniculata* (Burm.f.) has long been used in ayurvedic medicine through its anti-inflammatory properties. However, its protective effect of skin aging has not been studied in vitro. This study aimed to investigate the anti-aging effects of methanolic extract (ME), andrographolide (ANDRO), neoandrographolide (NEO), 14-deoxyandrographolide (14DAP) and 14-deoxy-11,12-didehydroandrographolide (14DAP11-12) on human dermal fibroblasts (HDFa) under pro-oxidant or pro-inflammatory condition. The in vitro anti-aging capacity of ME, ANDRO, NEO, 14DAP, and 14DAP11-12 (1, 2.5 and 5 µg/mL) was performed in HDFa. Oxidative stress and inflammation were induced by hydrogen peroxide and lipopolysaccharide/TNF-α, respectively. Reactive oxygen species (ROS) production was measured by the fluorescence of DCF-DA probe and cytokines were quantified by ELISA (IL6 and IL8) or RTqPCR (*TNF-*α). Procollagen type I production was determined by an ELISA. Our results showed a decrease in ROS production with ME and 14DAP at 5 µg/mL and 1 µg/mL, respectively. Furthermore, IL-6 production and *TNF-*α expression decreased under ANDRO and ME at 5 µg/mL. Our data indicated that ME and 14DAP protect from oxidative stress. Additionally, ME and ANDRO decreased an inflammation marker, IL-6. This suggests their potential natural treatment against skin damage. Hence, their applications could be of interest in cosmetics for preventing skin ageing.

## 1. Introduction

Skin aging is a complex and unavoidable process. It is divided into two categories: intrinsic aging and extrinsic aging. Intrinsic aging is mainly caused by cell senescence, while extrinsic aging is due to environmental factors such as UV [1,2]. Extrinsic UV-induced aging is called photoaging. It is associated with reactive oxygen species (ROS) production that exceeds the antioxidant defenses [3,4]. This imbalance causes oxidative stress in cells. Interestingly, ROS production activates the nuclear factor-kappaB (NF-κB) signaling pathway [5,6,7]. Briefly, an excess of ROS induced by UV radiation can activate the IkB kinase, which leads to the phosphorylation IkB and finally to the activation of NF-κB [8,9]. This activation induces pro-inflammatory cytokine secretion, such as interleukin-6 (IL-6), interleukin-8 (IL-8), and tumor necrosis factor-α (TNF-α) [10,11].

Moreover, NF-κB activation increases matrix metalloproteinases (MMPs) expression, leading to extracellular matrix degradation including collagen [12,13,14,15]. Collagen fibers are essential elements in the dermis architecture [16,17]. Both the quality and quantity of collagen are essential to the cutaneous mechanical properties [18,19,20,21]. In the dermis, fibroblasts synthesize collagen type I and III [22]. It is noted that this capacity drops with age [23,24]. Therefore, the low collagen production and the action of MMPs lead to sagging skin and the formation of wrinkles [25].

In order to reduce skin aging, synthetic antioxidants are widely used in cosmetic products. However, their long-term use may trigger toxic effects. Many studies have shown the interest of natural antioxidant and anti-inflammatory compounds against skin aging [26,27,28]. In addition, the bioactive molecules are less allergenic and very efficient [29]. In this study, we focused on *Andrographis paniculata* (Burm.f.) (synonym *Justicia paniculata*, common name “king of bitter”), which is part of the *Acanthaceae* family. It has been used in conventional medicine in Asia to treat inflammatory diseases such as respiratory diseases (influenza, cough, and bronchitis) and hepatitis [30,31].

**Figure 1:**
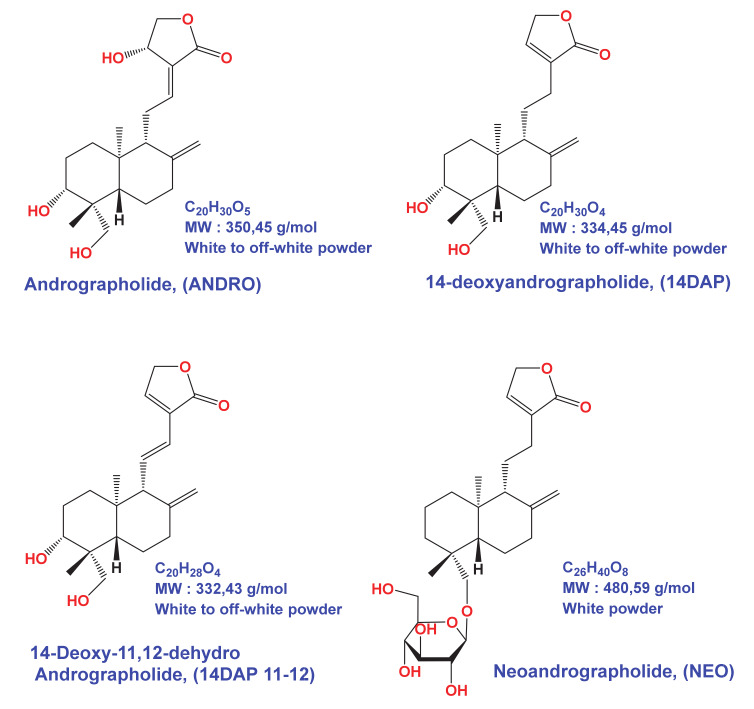
Andrographolide (ANDRO), 14DAP, 14DAP11-12 and neoandrographolide (NEO) molecular structures.

Methanol extract (ME) from the leaves of *Andrographis paniculata* has been shown to be anti-inflammatory [32], antioxidant [33,34] and antitumor [35]. The same extract showed a hepatoprotective [36] and antiviral effect [37]. In these extracts, many bioactive compounds are identified, including diterpenes [38]. In 2014, Sareer et al. showed that Andrographolide (ANDRO) is the main bioactive compound in the leaves of *Andrographis paniculata* [39]. To date, this molecule is known for its antiviral [40], anticancer [41], hepatoprotective [42], antioxidant [43] and anti-inflammatory [44] properties. Other diterpenoids are present in the aerial part of the plant: 14-deoxyandrographolide (14DAP) and 14-deoxy-11,12-didehydroandrographolide (14DAP11–12), neoandrographolide (NEO) (Figure 1). Antioxidant and anti-inflammatory effects of these derivatives have been reported in several publications [45,46,47,48,49,50,51].

However, the effect of these molecules on skin aging, notably on cells oxidation and inflammation, has not been explored. Herein, we aim to evaluate the potential effect of the methanolic extract of *Andrographis paniculata* (ME), ANDRO (MW: 350.45 g/mol), NEO (MW: 480.59 g/mol), 14DAP (334.45 g/mol), and 14DAP11-12 (332.43 g/mol), on human skin fibroblasts under oxidative stress or pro-inflammatory condition.

## 2. Materials and Methods

### 2.1. Chemicals

Andrographolide (ANDRO, MW 350.45 g/mL, Ref. 365645, ≥98% purity) were obtained from Sigma-Aldrich (Sigma-Aldrich, Saint-Louis, MO, USA). Neoandrographolide (NEO, MW 480,59 g/mL, Ref. MN11576, ≥98% purity), 14-Deoxyandrographolide (14DAP, MW 334,45 g/mL, Ref. FD139289, ≥95% purity), and 14-Deoxy-11,12-didehydroandrographolide (14DAP11-12, MW 332,43 g/mL, Ref. FD42724, 97% purity) were purchased from Carbosynth (Carbosynth, Compton, Berkshire, UK). The compounds were illustrated in Figure 1. The molecules were dissolved in dimethyl sulfoxide (DMSO) as a stock solution at 10  mg/mL and stored at −20 °C.

### 2.2. Preparation of A. paniculata Extract

The dried leaves of *Andrographis paniculata* were purchased from AYur-vana^®^. The leaf powder was extracted by maceration in methanol (15 mL/g) for 2 h at RT and then sonicated for 1 h in ice. The crude extract was filtered, and the methanol was evaporated overnight at RT. The extract was suspended in DMSO (0.6 g/mL) and filtered (methanolic extract ME)

### 2.3. HPLC Analysis

Methanolic extract was analyzed by reverse-phase HPLC using a Zorbax Eclipse XDB-C18 column 4.6 × 150 mm (Agilent) on an Agilent 1220 Infinity II LC System. The mobile phase was delivered at a rate of 1 mL/min, with a gradient from A (0.1% HCOOH in H_2_O) to B (0.1% HCOOH in CH_3_CN) (10% B for 4 min, 10% to 60% B in 10 min, 60% to 100% B in 2 min.). The column effluent was monitored at 250 nm.

### 2.4. Cell Culture

Human dermal fibroblasts, adult (HDFa) was obtain from Gibco (Gibco, Life Technologies Corp. C-013-5C, Carlsbad, CA). HDFa were cultured with DMEM (Sigma-Aldrich, Saint-Louis, MO, USA) supplemented with 10% heat-inactivated FBS (Sigma-Aldrich, Saint-Louis, MO, USA), 2% L-glutamine (Lonza, Basel, Switzerland) and 1% Penicillin-Streptomycin-Amphotericin B Mixture (Lonza, Basel, Switzerland). For the experiments, HDFa were used from passage P3 to passage P7. HDFa were seeded at a density of 5000 cells/cm² and maintained at 37 °C in 5% CO_2_. The medium was changed twice a week. Cell confluence at the time of experiment was approximately 80%.

### 2.5. Cell Treatment

Experimental group cells were treated with ME, ANDRO, NEO, 14DAP or 14DAP11-12. The concentration ranges used were 1, 2.5 or 5 µg/mL for ANDRO, NEO, 14DAP and 14DAP11-12, and equivalent to 1, 2.5 or 5 µg/mL of andrographolide for ME. The control cells were treated with 0.05% DMSO.

### 2.6. MTT Assay

Cell viability was assessed using a colorimetric assay that reduces MTT (Sigma-Aldrich, Saint-Louis, MO, USA) to formazan dye, producing a purple color. Briefly, HDFa was seeded in a 96-well plate at 8 × 10^3^ cells/well. After 24 h of incubation, cells were treated with the concentration range of ME, ANDRO, NEO, 14DAP and 14DAP11-12 for 24 h or 48 h. Then, 10% (w/v) of MTT solution (5 mg/mL) was added to each well and further incubated for 4 h at 37 °C, 5% CO_2_. The medium was removed, and the blue crystals were dissolved in 100 µL SDS-acidic-isopropanol solution (0.5% SDS; 80 mM HCl). The optical density (OD) of each well was measured at 450 nm using 620 nm reference with a microplate reader (Multiskan GO Microplate Spectrophotometer, Thermo Fisher Scientific Inc., Illkirch-Graffenstaden, France).The assay performed in 6 replicates of three independent experiments (*n* = 3).

### 2.7. Lactate Deshydrogénase Activity

Cell cytotoxicity was assessed by determining released lactate dehydrogenase (LDH) into the medium by damaged cells, using Pierce LDH Cytotoxicity Assay Kit (Thermo Fisher Scientific Inc., Illkirch-Graffenstaden, France). This method is based on the LDH-catalyzed reduction of pyruvate lactate by NADH. Cells were seeded in 96-well plates at a density of 1 × 10^4^ cells per well. After 24 h of incubation, cells were treated with the concentration range of ME, ANDRO, NEO, 14DAP and 14DAP11-12 for 24 h or 48 h. Briefly, equal amounts of culture supernatant were mixed with reaction mixture containing NADH. After 30 min at room temperature, the reaction was stopped by Stop Solution. The absorbance was measured with a microplate reader (Multiskan GO Microplate Spectrophotometer, Thermo Scientific) at 490 nm using 680 nm reference. LDH activity released in maximum LDH release by complete lysis of cells were determined. Data are presented as the percentage of LDH released into the medium relative to maximum LDH control. The assay performed in 6 replicates of three independent experiments (*n* = 3).

### 2.8. Intracellular Reactive Oxygen Species (ROS)

The intercellular production of ROS levels was determined using DCFH-DA (2′,7′-dichlorofluorescein diacetate) (Sigma-Aldrich, Saint-Louis, MO, USA). The permeable DCFH-DA is oxidized by ROS to the highly fluorescent compound 2′,7′-chlorofluorescein (DCF). HDFa were seeded in 96-well plates at 1.5 × 10^4^ cells/well. After 24 h of incubation, the medium was replaced by DMEM containing 25 µM DCFH-DA for 45 min at 37 °C. Then, DCFH-DA was removed, and the cells were washed with PBS. Afterwards, the cells were incubated with the concentration range of ME, ANDRO, NEO, 14DAP or 14DAP11-12 with or without 0.5 mM H_2_O_2_ (as free radical generator), for 1 h at 37 °C. Subsequently, fluorescence intensity per each well was detected using a microplate reader (EMax; Molecular Devices, Sunnyvale, CA), at an excitation wavelength of 485 nm and at an emission wavelength of 520 nm. The fluorescence intensity is directly proportional to the concentration of free radical compounds. The assay performed in 6 replicates of three independent experiments (*n* = 3).

### 2.9. Quantitative RT-PCR

HDFa were seeded in 6-well plates at 4.5 × 10^4^ cells/well, up to 80% confluence. Then, cells were pretreated with the concentration range of ME, ANDRO, NEO, 14DAP or 14DAP11-12 for 18 h and LPS (Sigma-Aldrich, Saint-Louis, MO, USA) was added in the medium at 10 µg/mL for an additional time of 6 h. Total RNA was isolated from cells using RNeasy Mini Kit (Qiagen, Hilden, Germany), following the manufacturer’s instructions. Nucleic acid concentration and purity were determined by μDrop™ plate (Thermo Fisher Scientific Inc.). One microgram of total RNA was retrotranscribed using QuantiTect^®^ Reverse Transcription kit (Qiagen, Hilden, Germany), following the manufacturer’s procedure. The reaction was performed according to the manufacturer’s instructions of QuanTitect^®^ SYBR Green Master Mix (Qiagen, Hilden, Germany). Quantitative PCR was performed by C1000^TM^ Thermal cycler (CFX96^TM^ Real-Time System, Bio-Rad, les Ulis, France), under the following conditions: 10 min 95 °C, followed by 40 cycles of 15 s 95 °C and 1 min 60 °C. Quantitative PCR reaction was performed using specific primers: human TNF-α (Invitrogen: forward, 5′-CTC TTC TGC CTG CTG CAC TT-3′; reverse, 5′ CAG CTT GAG GGT TTG CTA CA3′) and GAPDH (Qiagen cat. #QT00079247), as an internal control. Data was analyzed using 2^−ΔΔCT^ method. The assay performed in 2 replicates of three independent experiments (*n* = 3).

### 2.10. Measurement of IL-6 and IL-8 Secretion

HDFa were seeded in 24-well plates at 1 × 10^4^ cells/well up to 80% confluence and then further cultured in fresh DMEM containing ME, ANDRO, NEO, 14DAP or 14DAP11-12, with or without TNF-α (10 ng/mL; as cytokines generator) for 24 h. Supernatants were collected and used in analysis of newly secreted interleukins. IL-6 and IL-8 were quantified using a sandwich ELISA assay kit (Peprotech, Rock Hill, NJ, USA), according to the manufacturer’s protocol. The assay performed in 2 replicates of three independent experiments (*n* = 3).

### 2.11. Measurement of Procollagen Type I Secretion

HDFa were seeded in 24-well plates at 2.5 × 10^4^ cells/well overnight and then further cultured in fresh serum-free DMEM, with or without ME, ANDRO, NEO, 14DAP or 14DAP11-12 for 48 h. Collected cell-free supernatants were analyzed for the level of procollagen type I carboxy-terminal (PIP) by an ELISA kit (TaKaRa Bio Inc., Otsu, Japan), according to the manufacturer’s recommended protocol. The assay performed in 2 replicates of three independent experiments (*n* = 3).

### 2.12. Statistic Test

All data are presented as mean ± standard deviation (SD). Comparisons between groups were analyzed using GraphPad Prism software via ANOVA by Kruskal–Wallis statistic (Dunn’s multiple comparisons test). Difference with *p*-value < 0.05 was considered significant.

## 3. Results

### 3.1. Analysis of Methanolic Extract from Andrographis paniculata

**Figure 2:**
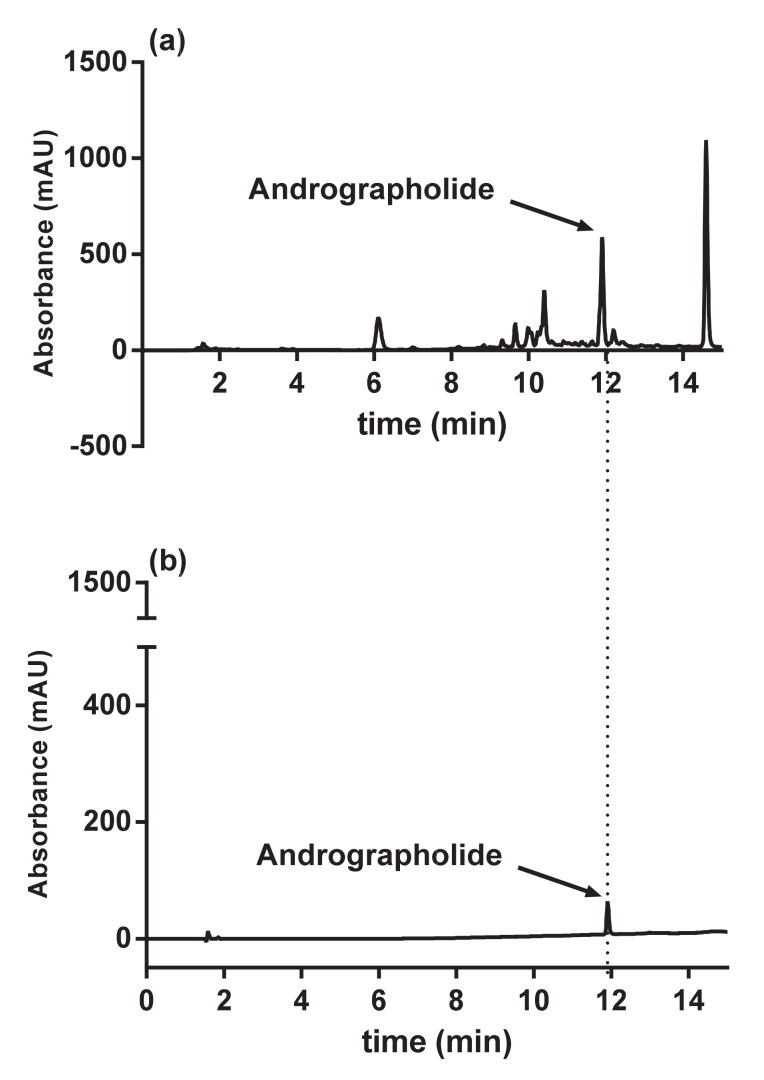
HPLC results of (**a**) andrographolide from methanolic extract of *Andrographis paniculata*; (**b**) andrographolide standard.

ANDRO was identified in our extract of *Andrographis paniculata*. ANDRO peaks from ME were identified at 11.9 min, by comparing the retention times obtained using the andrographolide standard. As shown in Figure 2, we detected a proportion of ANDRO in ME of 0.87%. We tested this extract according to the ANDRO concentration.

### 3.2. Cytotoxicity Assays

**Figure 3:**
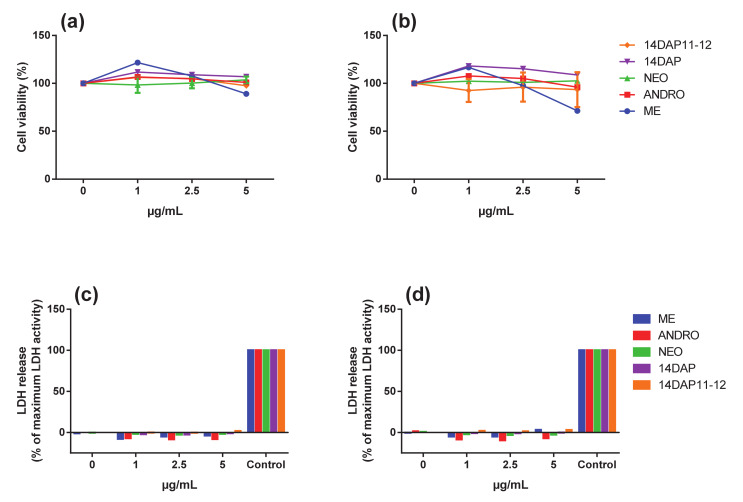
Effect of ME, ANDRO, NEO, 14DAP, and 14DAP11-12 on HDFa cytotoxicity. HDFa were treated with increasing concentration (1, 2.5 or 5 µg/mL) of ME, ANDRO, NEO, 14DAP, or 14DAP11-12 for 24 h (**a**,**c**) and 48 h (**b**,**d**). The control cells were treated with 0.05% DMSO. Cell viability and cell cytotoxicity were determined by an MTT assay (**a**,**b**) and a dosage of LDH release (**c**,**d**), respectively. The values mean ± S.D. * *p* < 0.05 were compared with control group, *n* = 3.

First, the cytotoxicity of ME, ANDRO, NEO, 14DAP, and 14DAP11-12 (1, 2.5, and 5 µg/mL) on HDFa was measured after 24 h and 48 h. Hence, metabolic activity was measured by an MTT assay and cell cytotoxicity was performed by a dosage of the LDH release. All treatments did not show cytotoxic effects on HDFa (Figure 3). Therefore, ME, ANDRO, NEO, 14DAP, and 14DAP11-12 were used in subsequent experiments at 1, 2.5 and 5 µg/mL.

### 3.3. Antioxidant Activity

**Figure 4:**
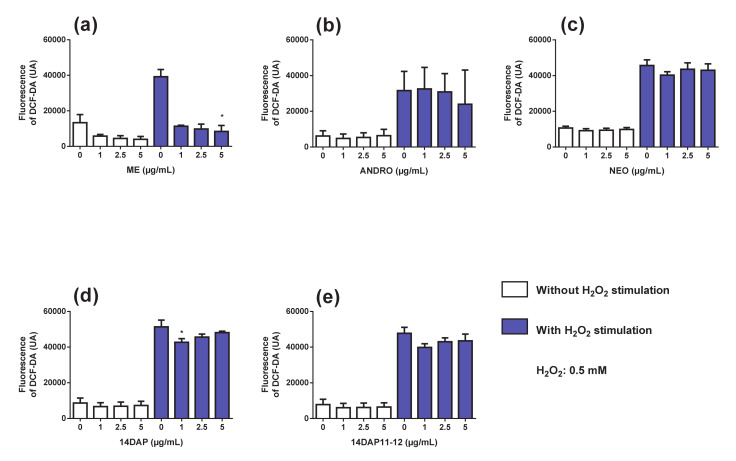
Effect of ME, ANDRO, NEO, 14DAP, and 14DAP11-12 on oxidative stress. HDFa were treated with increasing concentration (1, 2.5 or 5 µg/mL) of ME (**a**), ANDRO (**b**), NEO (**c**), 14DAP (**d**), or 14DAP11-12 (**e**) for 1 h. The control cells were treated with 0.05% DMSO. ROS production was induced by 0.5 mM H_2_O_2_ and free radical scavenging activity was done using a DCFH-DA probe. The values mean ± S.D. * *p* < 0.05 compared with control group, *n* = 3.

Next, we analyzed the effect of ME, ANDRO, NEO, 14DAP, and 14DAP11-12 on ROS production in H_2_O_2_-stimulated HDFa for 1 h. H_2_O_2_ caused an increase in intracellular ROS levels in HDFa compared to non-stimulated cells (Figure 4). This increase in ROS production under H_2_O_2_ pression was significantly reduced by ME treatment at 5 µg/mL (Figure 4a) and by 14DAP at 1 µg/mL (Figure 4d), compared to H_2_O_2_ condition (79% and 17% of decrease, respectively). In contrast, ANDRO, NEO and 14DAP11-12 had been unsuccessful in decreasing ROS levels (Figure 4b–e) under oxidative stress.

### 3.4. TNF-αExpression

**Figure 5:**
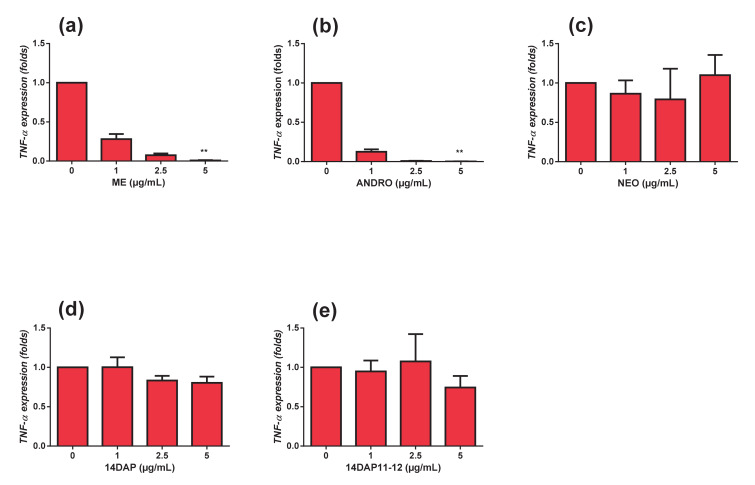
Effect of ME, ANDRO, NEO, 14DAP, and 14DAP11-12 on *TNF-α* expression under pro-inflammation condition. HDFa were treated with increasing concentration (1, 2.5 or 5 µg/mL) of ME (**a**), ANDRO (**b**), NEO (**c**), 14DAP (**d**), or 14DAP11-12 (**e**) for 24 h. The control cells were treated with 0.05% DMSO. Inflammation condition was induced by LPS (10 µg/mL) and *TNF-α* expression was determined by a RT-qPCR. The values mean ± S.D. * *p* < 0.05, ** *p* < 0.01 compared with control group, *n* = 3.

*TNF*-α expression was explored on HDFa pretreated with ME, ANDRO, NEO, 14DAP, and 14DAP11-12 for 18 h and then stimulated with LPS at 10 µg/mL for an additional 6 h. Under pro-inflammatory condition, we observed that ME and ANDRO at 5 µg/mL significantly decreased *TNF-α* mRNA level (Figure 5a,b). However, NEO, 14DAP, and 14DAP11-12 treatments did not lead to a decrease of *TNF-α* mRNA expression (Figure 5c–e).

### 3.5. Cytokine Secretions

As mentioned above, LPS was used to induce an inflammatory stress. We observed that HDFa treated by LPS showed an increase in IL-6 and IL-8 cytokines production. In these conditions, our treatments did not modify these increases (data not shown).

**Figure 6:**
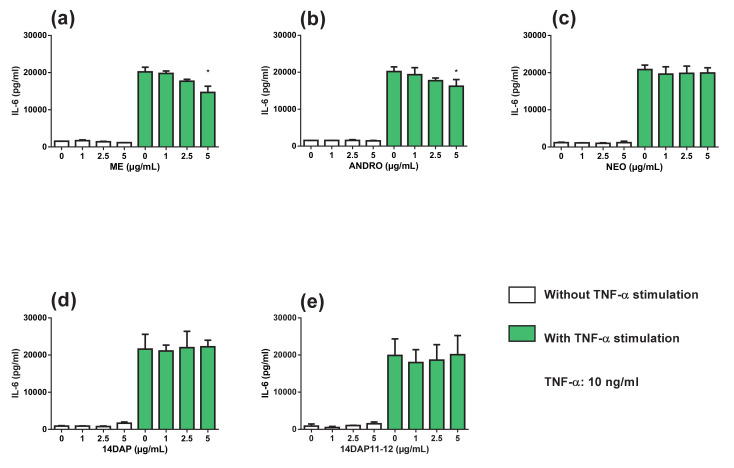
Effect of ME, ANDRO, NEO, 14DAP, and 14DAP11-12 on IL-6 secretion. HDFa were treated with increasing concentration (1, 2.5 or 5 µg/mL) of ME (**a**), ANDRO (**b**), NEO (**c**) 14DAP (**d**), or 14DAP11-12 (**e**) for 24 h. The control cells were treated with 0.05% DMSO. Inflammation was induced by TNF-α (10 ng/mL) and cytokine secretion was done using an ELISA assay. The values mean ± S.D. * *p* < 0.05 compared with control group, *n* = 3.

**Figure 7:**
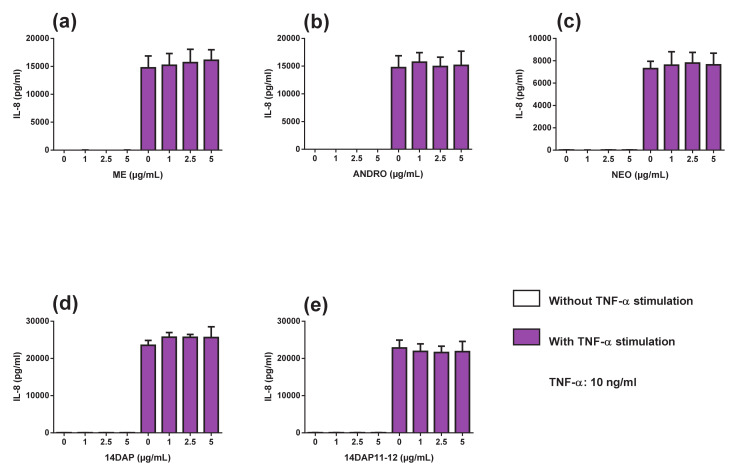
Effect of ME, ANDRO, NEO, 14DAP, and 14DAP11-12 on IL-8 secretion. HDFa were treated with increasing concentration (1, 2.5 or 5 µg/mL) of ME (**a**), ANDRO (**b**), NEO (**c**) 14DAP (**d**), or 14DAP11-12 (**e**) for 24 h. The control cells were treated with 0.05% DMSO. Inflammation was induced by TNF-α (10 ng/mL) and cytokines secretion was done using an ELISA assay. The values mean ± S.D. * *p* < 0.05 compared with control group, *n* = 3.

Thus, the inflammation was induced by another stimulation. We observed that TNF-α treatment led to an increase in IL-6 and IL-8 production in HDFa, compared to unstimulated cells (Figure 6 and Figure 7). ANDRO and ME significantly reduced IL-6 secretion at 5 µg/mL (20% and 27% of decrease, respectively) in inflammation condition (Figure 6a,b). However, NEO, 14DAP, and 14DAP11-12 did not modify IL-6 production (Figure 6c–e) under TNF-α pression. As regards IL-8 cytokine, our treatments did not modify the IL-8 secretion (Figure 7).

### 3.6. Pro-collagen Type I Production

**Figure 8:**
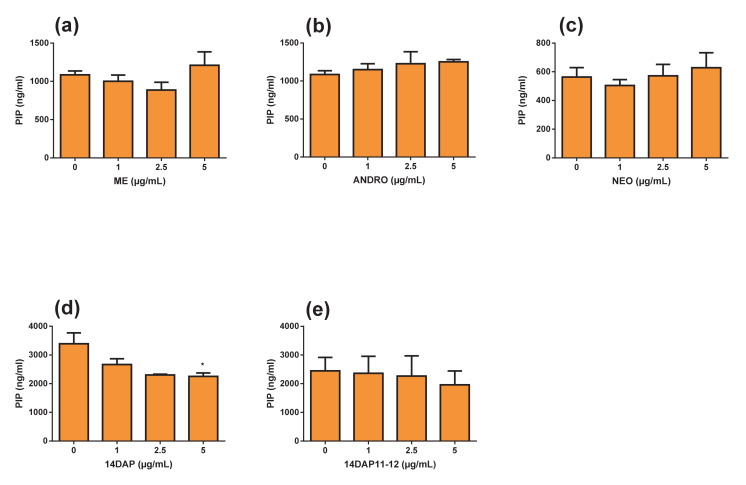
Effect of ME, ANDRO, NEO, 14DAP, and 14DAP11-12 on procollagen type I C-peptide (PIP) production. HDFa were treated with increasing concentration (1, 2.5 or 5 µg/mL) of ME (**a**), ANDRO (**b**), NEO (**c**) 14DAP (**d**), or 14DAP11-12 (**e**) for 48 h. PIP was determined by an ELISA. The values mean ± S.D. * *p* < 0.05, ** *p* < 0.01 compared with control group, *n* = 3.

Finally, we measured the pro-collagen type I synthesis in HDFa after incubation with ME, ANDRO, NEO, 14DAP, and 14DAP11-12 for 48 h (Figure 8). As shown in Figure 8, there was a significant decrease for 14DAP at 5 µg/mL (34% decrease).

## 4. Discussion

Inflammation and oxidative stress are often involved in extrinsic skin aging [52]. Many natural agents with anti-inflammatory and antioxidant properties are of interest for the skin anti-aging process [26,27,28]. In this study, we focused on the *Andrographis paniculata* plant. We have explored the effect of ME, ANDRO, NEO, 14DAP, and 14DAP11-12 on oxidative stress, inflammation and collagen production.

Oxidative stress is attributed to the cytokines production and the collagen fragmentation [3]. Therefore, regulating ROS levels seems to be an interesting strategy, by which natural compounds with antioxidant activity could be used. In the present study, ME with the bioactive compound ANDRO (comprising 0.87% of ANDRO) showed a high antioxidant effect in HDFa (Figure 4b), as seen in prior studies [32,53] The antioxidant propriety of 14DAP at 1 µg/mL in HDFa has also been confirmed (Figure 4b). Based on our findings, it would appear that ANDRO isn’t responsible for ROS decrease in HDFa stimulated with ME under H_2_O_2_ pression. According to Malahubban et al., *Andrographis paniculata* leaves extracted with methanol had a high phenolic content. Also, this extract had the highest levels of bioactive compounds, followed by ethanol, water and chloroform. A large quantity of alkaloids, saponins, flavonoids, tannins, terpenoids and steroids has been found, including the highest amount of ANDRO. The presence of phenols and other bioactive molecules may explain the ROS decrease in the results we obtain with ME. One or more active ingredients can act alone or in synergy in this extract. Interestingly, we did not observe a decrease in ROS production in our cells treated with ANDRO. However, many studies have shown that ANDRO reduces oxidative stress [54,55,56]. For example, ANDRO (0.625 µM and 2.5 µM) decreased ROS production in a primary culture of rat chondrocytes co-treated with H_2_O_2_ for 1h [57]. ANDRO may act differently depending on the cell type. In mouse skin exposed to UVs, Zhan et al. have shown that topical application of ANDRO increased the activity of SOD and CAT in [58]. These enzymes are essential to the mechanism of free radical elimination and consequently the cellular redox balance [59].

With increased age, the immune systems undergo multiple changes. These include a progressive increase of proinflammatory cytokines production such as IL-6, IL-8, and TNFα with age, thus producing chronic low-grade inflammation [60]. Fibroblasts are non-immune cells but these cells have the ability to produce proinflammatory cytokines and participating in local inflammatory responses [61]. Cytokines such as IL-6 accelerated the secretion of MMP-1 and MMP-3 [62,63]. On the other hand, TGF-β1 induced procollagen type I synthesis. However, IL-6 limited the TGF-β1 pathway in dermal fibroblasts [64,65], hence leading to collagen loss in the dermis. Thus, the application of anti-inflammatory compounds appears to be a strategy to prevent skin aging. Our results showed that in HDFa, treatment with ANDRO or ME at 5 µg/mL while undergoing TNFα-stimulation resulted in a decrease in IL-6 secretion (Figure 6a,b). Previously, Sheeja and Kuttan, reported low cytokine levels in mice treated for metastatic tumor with extract of *Andrographis paniculate* [66]. They showed that ANDRO regulates IL-6 production and the latter plays a key role in the inflammatory response of HDFa [67]. In our study, ME and ANDRO at 5 µg/mL showed a decrease of *TNF-α* expression in HDFa under inflammation (Figure 5a,b). Our results confirm recent reports, which have shown an inhibitory effect of ANDRO on TNF-α in inflammation [44,68,69,70]. According to our results, ANDRO inhibits LPS-mediated TNF-α and IL-6 production in RAW 264.7 cells [71]. In the skin, andrographolide sodium bisulfate significantly reduced the secretion of TNF-α (1.2 and 3.6 mg/mouse) and IL-6 (3.6 mg/mouse) in mice skin exposed to UV radiation [58]. Interestingly, we did not observe any inhibition of IL-8 secretion in HDFa treated with TNF-α (Figure 7). However, in a colorectal line HCT116 treated with TNF-α, ANDRO (5, 10 and 20 µM) restrained IL-8 in regards to mRNA, protein and promoter activity [72].

In intrinsic aging, collagen level decreases, whereas in extrinsic aging, collagen level increases and often fragmented [73]. Collagen is mainly responsible for dermal strength (Fisher, 2005). In the present study, only NEO at 5 µg/mL decreased the procollagen type I in HDFa (Figure 8). NEO application could be interesting to reduce changes in the organization of connective tissue in skin damaged by photoaging. Topical delivery of NEO in skin, formulation, and therapeutic concentration in the dermis remain poorly reported. Exploring the delivery of NEO in a formulation type emulsion which allows skin penetration is of interest. Zhan et al. showed that Andrographolide sodium bisulfate (1.2 and 3.6 mg/mouse) prevents UV-induced collagen damage by inhibiting UV-induced increases in MMP-1 and MMP-3 expression in mice skin [58]. In fact, Andrographolide and its derivatives are known to have other interesting therapeutic effects than the antioxidant. Other Pharmacological properties of andrographolide are numerous such as, anti-tumoral, anti-inflammatory, common cold, hepatoprotective, and antiviral [30].

## 5. Conclusions

In this study, ME and 14DAP treatments decreased ROS production under oxidative stress condition in dermal fibroblasts. Additionally, ME and ANDRO treatments decreased IL-6 secretion and TNF-α expression under inflammatory condition. Finally, 14DAP decreased pro-collagen type I secretion. Hence, *Andrographis paniculata* may prevent skin damage.

## Abbreviations

14DAP	14-deoxyandrographolide
14DAP11-12	14-deoxy-11,12-didehydroandrographolide
ANDRO	andrographolide
HDFa	human dermal fibroblasts, adult
IL-6	interleukin-6
IL-8	interleukin-8
LDH	lactate dehydrogenase
ME	methanolic extract
NEO	neoandrographolide
PIP	procollagen type I carboxy-terminal
ROS	reactive oxygen species
TNF-α	tumor necrosis factor-α
